# ALPK1 mutants causing ROSAH syndrome or Spiradenoma are activated by human nucleotide sugars

**DOI:** 10.1073/pnas.2313148120

**Published:** 2023-12-07

**Authors:** Tom Snelling, Anton Saalfrank, Nicola T. Wood, Philip Cohen

**Affiliations:** ^a^Medical Research Council Protein Phosphorylation and Ubiquitylation Unit, School of Life Sciences, University of Dundee, Dundee DD1 5EH, Scotland, United Kingdom

**Keywords:** ALPK1, ROSAH, spiradenoma, TIFA, nucleotide sugar

## Abstract

The atypical protein kinase ALPK1 is activated by the bacterial nucleotide sugar ADP-heptose and phosphorylates TIFA to switch on a signaling pathway that combats microbial infection. In contrast, ALPK1 mutations cause two human diseases: the ALPK1[T237M] and ALPK1[Y254C] mutations underlie ROSAH syndrome (retinal dystrophy, optic nerve oedema, splenomegaly, anhidrosis, and migraine headache), while the ALPK1[V1092A] mutation accounts for 45% of spiradenoma and 30% of spiradenocarcinoma cases studied. In this study, we demonstrate that unlike wild-type (WT) ALPK1, the disease-causing ALPK1 mutants trigger the TIFA-dependent activation of an NF-κB/activator protein 1 reporter gene in the absence of ADP-heptose, which can be suppressed by either of two additional mutations in the ADP-heptose binding site that prevent the activation of WT ALPK1 by ADP-heptose. These observations are explained by our key finding that although ALPK1[T237M] and ALPK1[V1092A] are activated by bacterial ADP-heptose, they can also be activated by nucleotide sugars present in human cells (UDP-mannose, ADP-ribose, and cyclic ADP-ribose) which can be prevented by disruption of the ADP-heptose binding site. The ALPK1[V1092A] mutant was also activated by GDP-mannose, which did not activate ALPK1[T237M]. These are new examples of disease-causing mutations permitting the allosteric activation of an enzyme by endogenous molecules that the WT enzyme does not respond to. We propose that the loss of the specificity of ALPK1 for bacterial ADP-heptose underlies ROSAH syndrome and spiradenoma/spiradenocarcinoma caused by ALPK1 mutation.

## Significance

Mutations in the atypical protein kinase ALPK1 (alpha-protein kinase 1) cause two human diseases, ROSAH syndrome and spiradenoma/spiradenocarcinoma. In this study, we make the unexpected finding that in contrast to wild-type ALPK1, which is activated specifically by bacterial ADP-heptose, the disease-causing ALPK1 mutants are not only activated by ADP-heptose but also by several nucleotide sugars present in human cells, explaining how these diseases can arise independently of bacterial infection. These are new examples of disease caused by loss of specificity of an enzyme for its allosteric activator.

Alpha-protein kinase 1 (ALPK1) is one of the six members of the alpha family of atypical protein kinases, which display no similarity in amino acid sequence to any of the other families of human kinases (1). In 2018, ALPK1 was identified as a key component of a previously unrecognized innate immune signalling pathway in which this protein kinase is activated by the bacterial nucleotide sugar adenosine diphosphate-L-glycero-β-D-manno-heptose (ADP-L,D-heptose) or ADP-D,D-heptose (2). Since ADP-L,D-heptose and ADP-D, D-heptose activate ALPK1 similarly (2, 3), only ADP-D,D-heptose was used in this study (hereafter, simply referred to as ADP-heptose).

**Fig. 1.**
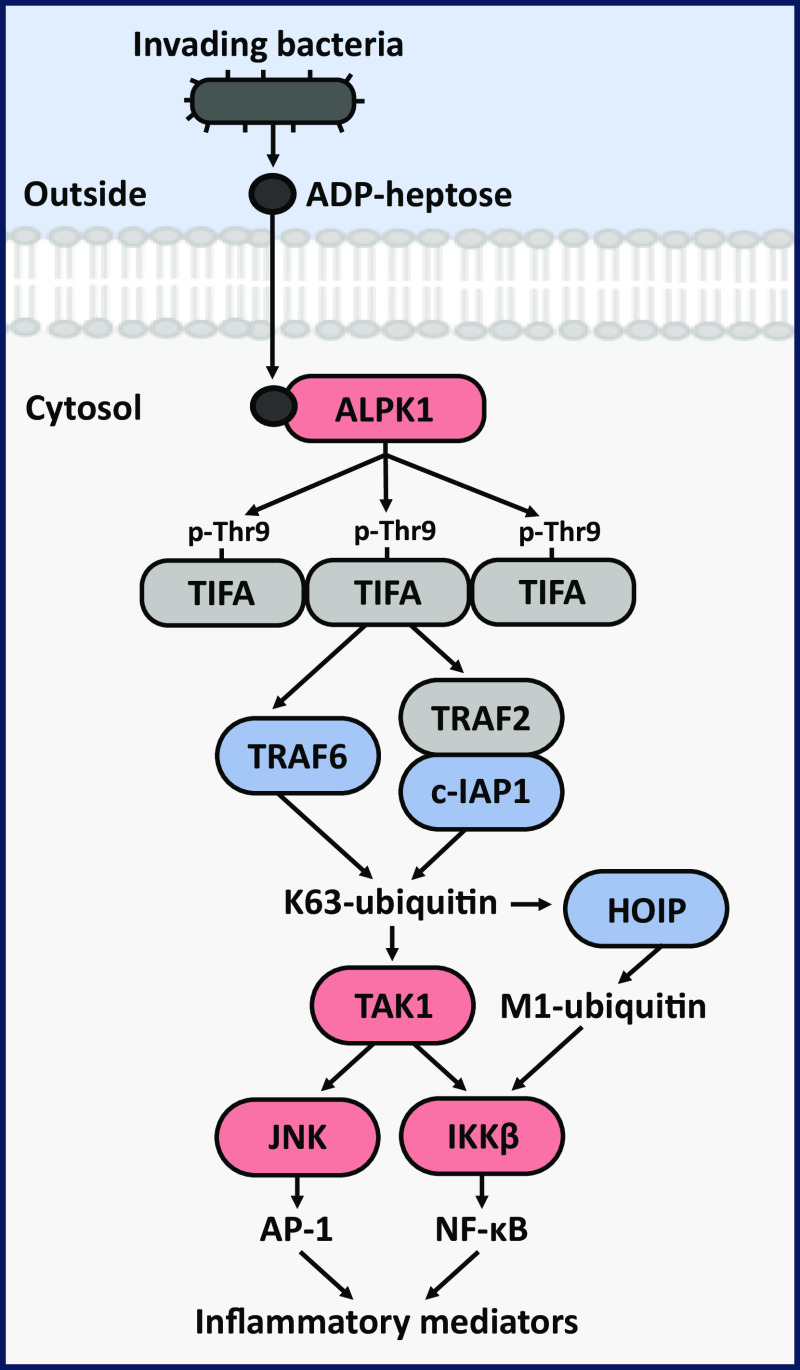
Outline of the ADP-heptose-ALPK1-TIFA signalling pathway. The bacterial nucleotide sugar ADP-heptose crosses the plasma membrane of human cells, where it binds to and activates the cytosolic human protein kinase ALPK1, allowing it to phosphorylate TIFA at Thr9. This induces the polymerisation of TIFA and the recruitment of the E3 ligases c-IAP1 (recruited by TRAF2) and TRAF6, which produce the Lys63-linked ubiquitin chains (K63-ubiquitin) required to activate the TAK1 kinase complex (3). TAK1 then activates JNK and the IKKβ component of the canonical IKK complex. IKKβ activation also requires the formation of Met1-linked ubiquitin chains (M1-ubiquitin) produced by the E3 ligase HOIP, a component of the LUBAC. How HOIP is recruited to the signalling complex is not fully understood, but the Npl4 zinc finger domain of HOIP interacts selectively with Lys63-linked ubiquitin oligomers (9). Once activated, JNK and IKKβ activate the transcription factors AP-1 and NF-κB, respectively, switching on the transcription of genes encoding inflammatory mediators. E3 ligases are highlighted in blue, protein kinases in pink, and noncatalytic components in grey.

The binding of ADP-heptose to an N-terminal domain of ALPK1 is thought to induce a conformational change that activates the C-terminal kinase domain, enabling ALPK1 to phosphorylate tumour necrosis factor (TNF) receptor-associated factor (TRAF)-interacting protein with forkhead-associated (FHA) domain (TIFA) at Thr9 (2). This induces the interaction of phosphorylated Thr9 with the FHA domain of another TIFA molecule, leading to the “head-to-tail” polymerization of TIFA into “TIFAsomes”, which recruit and activate three different E3 ubiquitin ligases, namely TRAF6, cellular inhibitor of apoptosis 1 (c-IAP1) (which is recruited by TRAF2) and LUBAC (the linear ubiquitin assembly complex) (2, 3). TRAF6 and c-IAP1 generate the Lys63-linked ubiquitin chains required to recruit and activate the transforming growth factor β-activated kinase 1 (TAK1) complex (3), which activates mitogen-activated protein kinase (MAPK) cascades that lead to the activation of p38 MAPKs and c-Jun N-terminal kinases (JNK). One role of JNK is to activate activator protein 1 (AP-1) transcription factor complexes (4). Another function of TAK1 is to activate the canonical IκB kinase (IKK) complex, which is facilitated by the LUBAC-catalysed formation of Met1-linked ubiquitin chains that interact with NEMO (NF-κB essential modulator), a regulatory subunit of the IKK complex (5–7). One of the key functions of the canonical IKK complex is to activate the transcription factor NF-κB (nuclear Factor kappa-light-chain-enhancer of activated B cells) (8). A schematic outlining the ALPK1 signalling pathway is presented (Fig. 1).

Mutations in ALPK1 were recently found to cause two human diseases. The mutation of Thr237 to Met [so far identified in 66 patients from 29 unrelated families (10–15)] and the mutation of Tyr254 to Cys [so far identified in only 1 patient (12)] cause an autosomal dominant disease termed ROSAH syndrome [Retinal dystrophy, Optic nerve oedema, Splenomegaly, Anhidrosis (the inability to sweat) and migraine Headache]. These patients usually present in the clinic with failing eyesight or severe abdominal pain caused by a massive increase in spleen size, but the exact phenotype varies and can include autoinflammatory conditions, such as arthritis. Interestingly, Thr237 and Tyr254 are both located within the N-terminal domain of ALPK1 containing the ADP-heptose binding site, but only Thr237 lies within the ADP-heptose binding site itself (2).

Spiradenomas are a group of neoplasms, usually observed as abnormal growths on the head, neck, and upper body, which may originate from sweat glands or from the bulges at the base of hair follicles (16). Although predominantly benign, they can sometimes undergo malignant transformation to spiradenocarcinomas, which have high rates of mortality even after surgery, chemotherapy and radiation (17). Spiradenocarcinomas can also arise de novo without the prior formation of spiradenomas. Remarkably, in a cohort of 30 unrelated patients with spiradenoma or spiradenocarcinoma, 7 of the 16 spiradenoma patients and 4 of the 14 spiradenocarcinoma patients were found to have the same Val1092 to Ala mutation located within the protein kinase domain of ALPK1 (18).

It is not known why or how ALPK1[T237M], ALPK1[Y254C], and ALPK1[V1092A] cause two such different human diseases. Here, we report that, in contrast to wild-type (WT) ALPK1, the reexpression of these three mutants in ALPK1 knockout (KO) human embryonic kidney 293 (HEK293) cells activated NF-κB/AP-1-dependent gene transcription in the absence of ADP-heptose, but this ADP-heptose-independent activity was nevertheless abolished (ALPK1[T237M] and ALPK1[Y254C]) or reduced (ALPK1[V1092A]) by further point mutations that prevent the activation of ALPK1 by ADP-heptose. These observations led us to identify nucleotide sugars present in human cells that cause activation of the disease-causing mutants of ALPK1, but not the WT protein.

## Results

### The Disease-Causing ALPK1 Mutants Induce NF-κB and AP-1-Dependent Gene Transcription in HEK293 Cells in the Absence of ADP-Heptose.

**Fig. 2.**
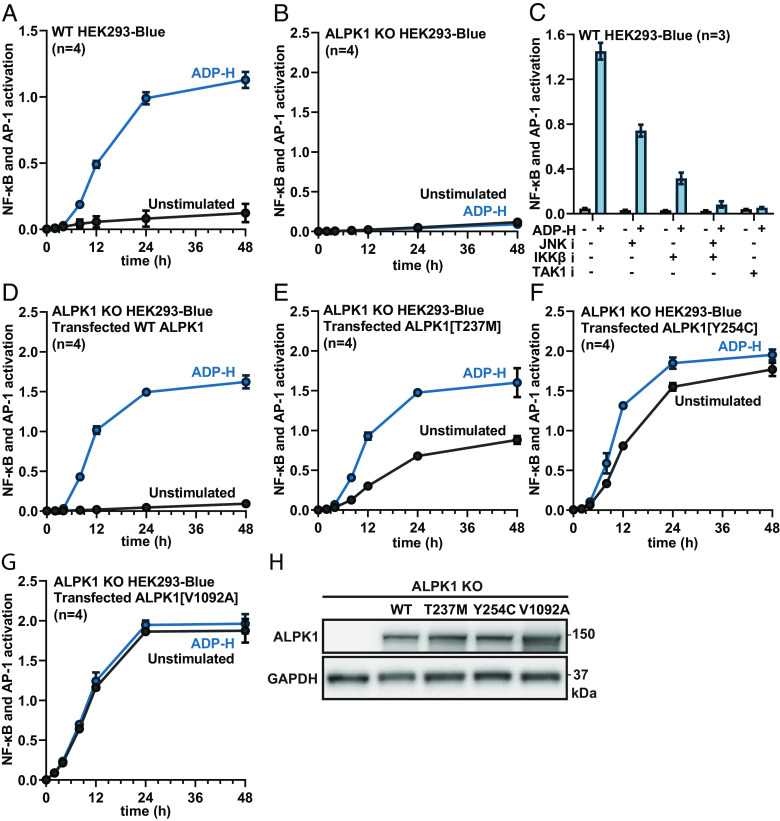
ADP-heptose-dependent and -independent NF-κB/AP-1 gene transcription in cells expressing WT ALPK1 and disease-causing mutants of ALPK1. (*A* and *B*) WT (*A*) or ALPK1 KO HEK293-Blue cells (*B*) were stimulated in the absence (black line) or presence (blue line) of ADP-heptose (ADP-H) and the activation of NF-κB/AP-1-dependent gene transcription measured after the times indicated (*Methods*). (*C*) WT cells were incubated for 1 h with 5 µM NG25 (TAK1 inhibitor, denoted TAK1 i), 10 µM BI605906 (IKKβ inhibitor, denoted IKKβ i), 5 µM JNK-IN-8 (JNK inhibitor, denoted JNK i) or a combination of 10 µM BI605906 and 5 µM JNK-IN-8 (IKKβ i + JNK i), as indicated. The cells were then incubated with (blue bars) or without (grey bars) ADP-H and the activation of gene transcription measured after 24 h. (*D*–*G*) ALPK1 KO cells were transfected with a plasmid encoding WT ALPK1 (*D*), ALPK1[T237M] (*E*), ALPK1[Y254C] (*F*) or ALPK1[V1092A] (*G*) as indicated and 24 h later were analysed as in *B*. (*H*) Cell extracts (15 µg of protein) from ALPK1 KO cells transfected with empty vector (lane 1), FLAG-tagged WT ALPK1 (lane 2), or the indicated FLAG-tagged ALPK1 mutant (lanes 3–5) were subjected to SDS-PAGE and immunoblotted using the antibodies indicated. (*A*–*G*) The results are expressed as mean ± SD from an experiment using three or four dishes of independently transfected cells and the experiment was repeated twice with similar results each time.

We studied ALPK1 activation in HEK293-Blue cells that contain a synthetic gene encoding secreted embryonic alkaline phosphatase (SEAP) under the control of NF-κB and AP-1 promoters (*Methods*). ADP-heptose stimulated the secretion of SEAP into the culture medium of the parental cell line expressing endogenous WT ALPK1, which was drastically reduced if ADP-heptose was omitted (Fig. 2*A*). The weak basal level of secretion was also observed in ALPK1 KO cells incubated with or without ADP-heptose, indicating that it was independent of ALPK1 activity (Fig. 2*B*). The ADP-heptose-stimulated secretion of SEAP was partially inhibited by the JNK inhibitor JNK-IN-8, partially inhibited by the IKKβ inhibitor BI605906, and completely prevented when both inhibitors were combined (Fig. 2*C*). The TAK1 inhibitor NG25, which prevents both JNK and IKKβ activation (3), also prevented the secretion of SEAP (Fig. 2*C*). These experiments established that, as expected, ADP-heptose stimulated the secretion of SEAP from these reporter cells via activation of the transcription factors AP-1 and NF-κB.

We next expressed FLAG-tagged WT ALPK1 or the three disease-causing mutants of ALPK1 into ALPK1 KO HEK293-Blue cells. The secretion of SEAP induced by ADP-heptose after 24 h was similar in cells expressing WT ALPK1, ALPK1[T237M], ALPK1[Y254C], or ALPK1[V1092A] (Fig. 2 *D*–*G*). In contrast, when ADP-heptose was omitted, SEAP secretion was drastically reduced in cells expressing WT ALPK1 as expected (Fig. 2*D*), but SEAP secretion from the cells expressing the ALPK1[T237M] (Fig. 2*E*) and ALPK1[Y254C] (Fig. 2*F*) mutants remained significantly elevated, and SEAP secretion from the cells expressing ALPK1[V1092A] was similar to that observed in the presence of ADP-heptose (Fig. 2*G*). These differences were not explained by variation in the relative levels of expression of these mutants, which were similar to WT ALPK1 (Fig. 2*H*). Other investigators have also reported that ALPK1[T237M], ALPK1[Y254C], and ALPK1 [V1092A] can activate NF-κB-dependent gene transcription or IL-8 secretion in HEK293 cells in the absence of ADP-heptose (12, 18), leading to the suggestion that these ALPK1 mutants are constitutively active (i.e., that they do not require an allosteric activator).

The ADP-heptose-independent activity of the three disease-causing ALPK1 mutants could have arisen by several mechanisms. First, the mutations may have caused ALPK1 to stimulate NF-κB/AP-1-dependent gene transcription via a distinct TIFA-independent pathway. Second, the mutations may have induced conformational changes that generated constitutively active forms of ALPK1. Third, the mutations may have permitted the activation of ALPK1 by one or more endogenous human metabolites. We therefore performed additional experiments to try and distinguish between these possibilities.

### ADP-Heptose-Independent Gene Transcription Requires the Protein Kinase Activity of ALPK1, the Expression of TIFA, and an Intact ADP-Heptose Binding Site.

**Fig. 3.**
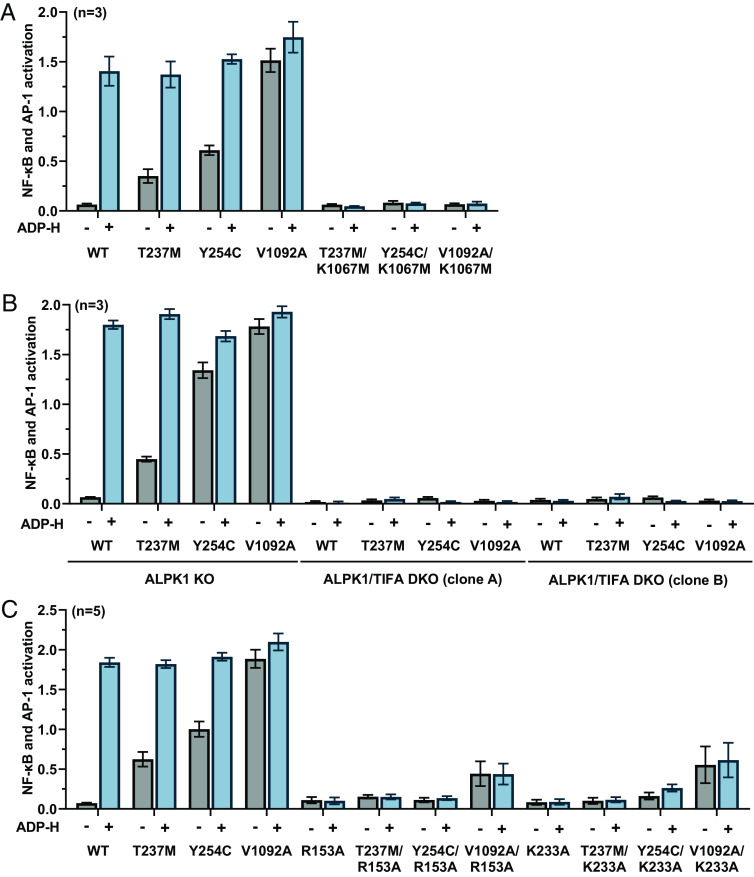
The cellular activity of ALPK1 disease mutants in the absence of ADP-heptose is dependent on the kinase activity of ALPK1, the expression of TIFA and an intact ADP-heptose binding site. (*A*) ALPK1 KO cells were transfected with plasmids encoding WT ALPK1 or the indicated ALPK1 mutants and 24 h later incubated with (blue bars) or without (gray bars) ADP-H and the activation of gene transcription measured after a further 24 h (*Methods*). (*B*) ALPK1 KO or two different clones of ALPK1/TIFA DKO cells (clones A and B) were transfected with the different plasmids indicated and analysed as in *A*. (*C*) As in *A*, but transfection was performed with the different plasmids indicated. (*A*–*C*) The results are expressed as mean ± SD from an experiment using three or five dishes of independently transfected cells and the experiment was repeated twice with similar results each time.

The mutation of Lys1067 to Met generates a catalytically inactive mutant of ALPK1 (2, 3). We found that double mutants in which each disease-causing mutation was combined with Lys1067Met were unable to induce gene transcription when expressed in ALPK1 KO HEK293-Blue cells (Fig. 3*A*). We next compared the effect of re-expressing the WT and mutant forms of ALPK1 in both ALPK1 KO cells and two independently isolated clones of ALPK1/TIFA double KO (DKO) cells. These experiments revealed that each ALPK1 mutant required the expression of TIFA to stimulate gene transcription in the absence or presence of ADP-heptose (Fig. 3*B*). Taken together, these results demonstrated that the disease-causing mutants required both ALPK1 catalytic activity and the expression of TIFA to induce gene transcription in HEK293-Blue cells, irrespective of whether ADP-heptose was present in the culture medium.

In order to distinguish between constitutive activity and activation by one or more endogenous human metabolites, we next generated double mutants in which the T237M, Y254C, and V1092A mutations were combined with the mutation of either Arg153 or Lys233 to Ala within the ADP-heptose binding site, which prevent the activation of WT ALPK1 by ADP-heptose (2). Arg153 and Lys233 form several polar interactions with ADP-heptose, which would be abolished by their mutation to Ala (*SI Appendix*, Fig. S1). Like the ALPK1[R153A] and ALPK1[K233A] mutants, the double mutants T237M/R153A, T237M/K233A, Y254C/R153A, and Y254C/K233A had little or no activity in cells stimulated with ADP-heptose but, surprisingly, they also blocked the activation of these disease-causing mutants in cells in the absence of ADP-heptose (Fig. 3*C*). These observations raised the possibility that the ROSAH-causing mutants of ALPK1 were being activated in HEK293 cells by endogenous human metabolite(s) (i.e., distinct from bacterial ADP-heptose) that, nevertheless, exerted their effect(s) by engaging the ADP-heptose binding site.

The cellular activity of the ALPK1[V1092A] mutant in the absence of ADP-heptose was reduced considerably when combined with the R153A or K233A mutation but was not abolished, in contrast to the two ROSAH-causing mutants (Fig. 3*C*). These results indicated that the ALPK1[V1092A] mutant possessed low constitutive activity in cells in the absence of ADP-heptose, which could be greatly enhanced by the binding of an unknown endogenous metabolite(s).

### Nucleotide Sugars Present in Human Cells Activate the Disease-Causing Mutants of ALPK1 In Vitro.

It has been reported that ALPK1 can be activated more efficiently by uridine diphosphate (UDP)-heptose than by ADP-heptose (19). UDP-heptose is not known to be present in any bacterial or mammalian cell, but this observation led us to examine whether UDP-sugar derivatives that are present in human cells could activate WT and mutant ALPK1. We also tested whether ADP-based nucleotide sugars or guanosine diphosphate (GDP)-α-D-mannose, which are also present in human cells, were activators of ALPK1 (20). These molecules were studied by assaying ALPK1 in vitro using GST-TIFA as a substrate (*Methods*).

**Fig. 4.**
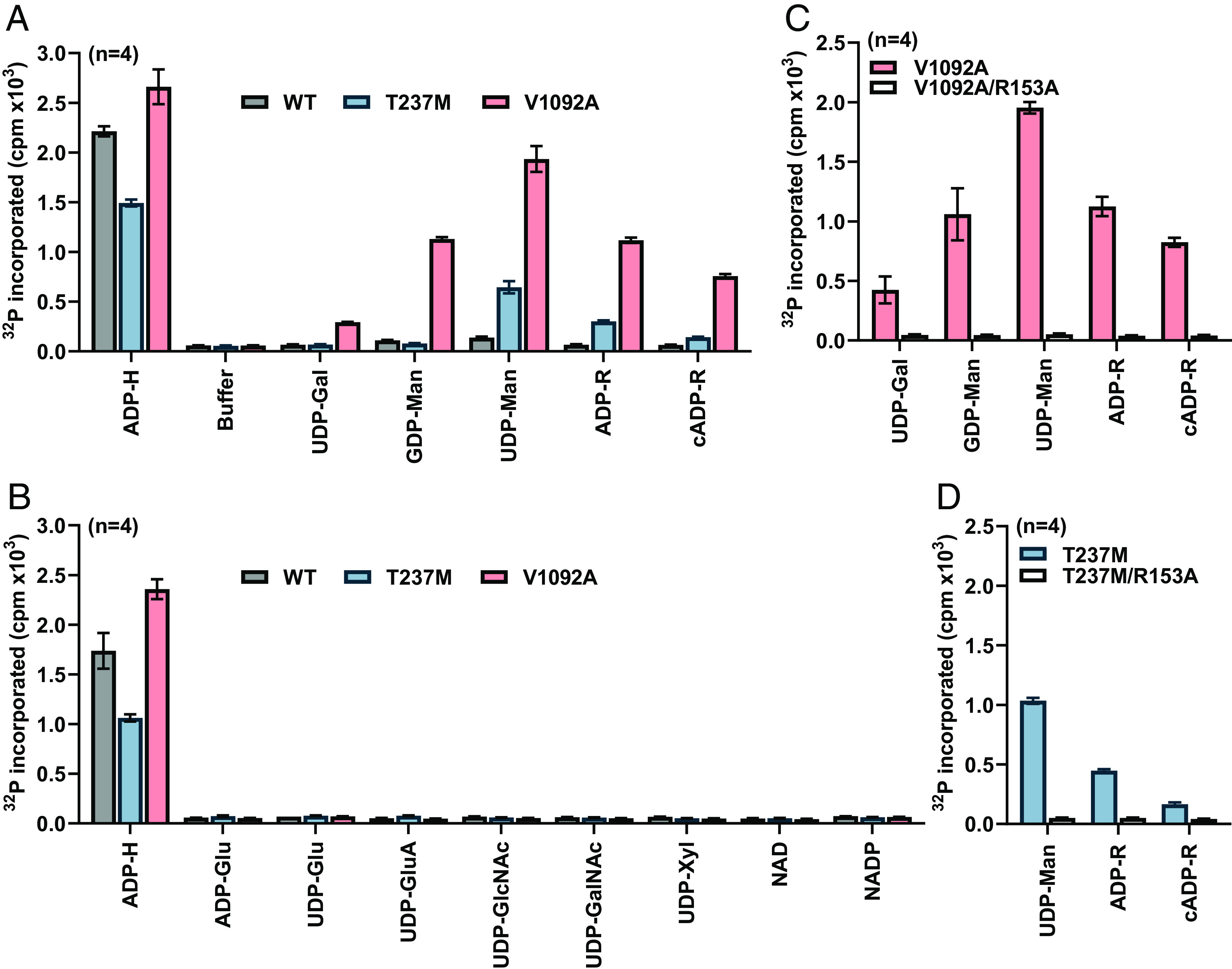
ALPK1 disease mutants, but not the WT protein, are activated by human nucleotide sugars in vitro. (*A* and *B*) FLAG-tagged WT ALPK1 (WT, gray bars), ALPK1[T237M] (T237M, blue bars) or ALPK1[V1092A] (V1092A, pink bars) were immunoprecipitated from cell extracts and assayed for 20 min in the absence or presence of ADP-heptose (1 µM) or the nucleotide sugars indicated (100 µM) and the incorporation of ^32^P-radioactivity into GST-TIFA quantified (*Methods*). (*C*) As in *A* and *B*, except that the activity of ALPK1[V1092A] was compared to ALPK1[V1092A/R153A] (V1092A/R153A, unfilled bars). (*D*) As in *A* and *B*, except that the activity of ALPK1[T237M] was compared to ALPK1[T237M/R153A] (T237M/R153A, unfilled bars). (*A*–*D*) The nucleotide sugars are abbrievated as follows: ADP-heptose (ADP-H), UDP-galactose (UDP-Gal), GDP-mannose (GDP-Man), UDP-mannose (UDP-Man), ADP-ribose (ADP-R), cyclic ADP-ribose (cADP-R), ADP-glucose (ADP-Glu), UDP-glucose (UDP-Glu), UDP-glucuronate (UDP-GluA), UDP-N-acetyl-glucosamine (UDP-GlcNAc), UDP-N-acetyl-galactosamine (UDP-GalNAc), and UDP-xylose (UDP-Xyl). The results are expressed as mean ± SEM from a total of 4 kinase assays (two experiments, each performed in duplicate).

Similar to WT ALPK1, ALPK1[T237M] and ALPK1[V1092A] had negligible activity in vitro without ADP-heptose and were activated strongly by ADP-heptose. However, the activity of ALPK1[T237M] was consistently lower than that of WT ALPK1, whereas the activity of ALPK1[V1092A] in the presence of ADP-heptose was consistently higher (Fig. 4 *A* and *B*).

Strikingly, both ALPK1[T237M] and ALPK1[V1092A] were activated by UDP-α-D-mannose, ADP-D-ribose and to a lesser extent by cyclic ADP-D-ribose, which did not activate WT ALPK1 (ADP-D-ribose, cyclic ADP-D-ribose) or only activated WT ALPK1 slightly (UDP-α-D-mannose) (Fig. 4*A*). Additionally, ALPK1[V1092A] was strongly activated by GDP-α-D-mannose, which did not affect ALPK1[T237M] and barely activated WT ALPK1 (Fig. 4*A*). ALPK1[V1092A] was also activated weakly by UDP-α-D-galactose, which did not affect either ALPK1[T237M] or WT ALPK1 (Fig. 4*A*). Six other nucleotide sugars and two dinucleotides tested did not activate the WT or mutant forms of ALPK1 (Fig. 4*B*).

To investigate whether the nucleotide sugar activators of the ALPK1 mutants were acting by engaging the ADP-heptose binding site, we studied double mutants in which each disease-causing mutation was combined with the R153A mutation. We found that combining the ALPK1[R153A] mutation with the spiradenoma mutant (Fig. 4*C*) or the ROSAH mutant (Fig. 4*D*) did indeed prevent activation by UDP-α-D-mannose, GDP-α-D-mannose, ADP-D-ribose, cyclic ADP-D-ribose, and UDP-α-D-galactose in vitro. This implied that these mammalian nucleotide sugars do bind to the ADP-heptose binding site, a result consistent with the cell-based assays (Fig. 3*C*).

Two of the nucleotide sugars that activated both ALPK1[T237M] and ALPK1[V1092A], namely UDP-α-D-mannose and ADP-D- ribose, were analyzed in more detail. The concentrations of UDP-α-D-mannose required for half-maximal activation of ALPK1[T237M] and ALPK1[V1092A] were 2.0 µM and 17 µM, respectively (*SI Appendix*, Fig. S2*A*), and the concentrations of ADP-D-ribose needed for half-maximal activation of these mutants were 7.1 µM and 19 µM, respectively (*SI Appendix*, Fig. S2*B*).

The immunoprecipitated FLAG-ALPK1[Y254C] mutant was almost devoid of activity in vitro (*SI Appendix*, Fig. S3*A*), although it was present at a similar level to the other ALPK1 mutants and WT ALPK1 (*SI Appendix*, Fig. S3*B*). This indicated that although ALPK1[Y254C] is active in HEK293-Blue cells (Fig. 2*F*), it rapidly loses activity during purification from cell extracts. For this reason, it was not possible to study the effects of nucleotide sugars on ALPK1[Y254C] in vitro.

## Discussion

A key observation made in the present study was that the ALPK1[R153A] and ALPK1[K233A] mutations, which are located within the ADP-heptose binding domain (*SI Appendix*, Fig. S1) and prevent the activation of ALPK1 by ADP-heptose (2) (Fig. 3*C*), also prevented the ROSAH-causing ALPK1[T237M] and ALPK1[Y254C] mutants from activating NF-κB/AP-1-dependent gene transcription in the absence of ADP-heptose in cells (Fig. 3*C*). These observations led us to identify UDP-α-D-mannose, ADP-D-ribose, and cyclic ADP-D-ribose as activators of the ALPK1[T237M] mutant in vitro (Fig. 4*A*). The spiradenoma-causing ALPK1[V1092A] mutant was not only activated by these three nucleotide sugars but also by GDP-α-D-mannose (and weakly by UDP-α-D-galactose) (Fig. 4*A*). These are new examples of disease-causing mutations permitting the abnormal activation of an enzyme by allosteric effectors that the WT enzyme does not respond to (ADP-D-ribose, cyclic ADP-D-ribose and UDP-α-D-galactose) or hardly responds to (UDP-α-D-mannose and GDP-α-D-mannose). However, we have not yet excluded the possibility that mammalian nucleotide sugars also bind to WT ALPK1 but without activating it. Direct binding studies will be required to resolve this issue.

GDP-α-D-mannose is the major substrate used to mannosylate glycoproteins in mammalian cells and tissues (21). UDP-α-D-mannose has also been detected in mammalian tissues and its formation is enhanced when mammalian cells are incubated with mannose. It is thought to be involved in protein glycosylation reactions but its physiological and pathological roles are still poorly understood and its concentration in cells is lower than that of GDP-α-D-mannose (22). ADP-D-ribose can be formed from either poly(ADP-D-ribose) by poly(ADP-ribose) glycohydrolase and ADP-ribosylhydrolase 3 (23) or from cyclic ADP-D-ribose by CD38 or SARM1, which are the enzymes that also form cyclic ADP-D-ribose from nicotinamide adenine dinucleotide (NAD) (24–26). However, we cannot exclude the possibility that ALPK1 mutants are also activated by other human nucleotide sugars that have not yet been tested.

Thr237 is located within the ADP-heptose binding site of WT ALPK1, where it forms a hydrogen bond with a hydroxyl group of the sugar moiety of ADP-heptose (*SI Appendix*, Fig. S1). The mutation of Thr237 to Met would prevent the formation of this hydrogen bond and might explain why the mutant becomes responsive to some mammalian nucleotide sugars. In contrast, Val1092 is located within the kinase domain of WT ALPK1, which is remote from the ADP-heptose binding site in the linear amino acid sequence of the protein kinase. Structural predictions using AlphaFold2 (27) suggest that the catalytic kinase domain interacts with the ADP-heptose binding domain (*SI Appendix*, Fig. S4) and, consistent with this notion, the coexpression of plamids encoding ALPK1[1-473] (containing the ADP-heptose binding domain) and ALPK1[959-1244] (comprising the catalytic kinase domain), produce a functional complex that is ADP-heptose-dependent (2). The interaction between these two domains may help to explain why mutation of Val1092 to Ala in the kinase domain affects the ADP-heptose binding pocket in a way that permits activation by mammalian nucleotide sugars. However, elucidation of the exact molecular mechanism will require the structure of the full-length protein and the structural transitions induced by this mutation to be elucidated.

ROSAH leads to retinal degeneration and anhidrosis (the inability to sweat) and spiradenomas originate from either sweat glands or the bulbs of hair follicles (see Introduction). It is therefore possible that the cells in which these diseases develop express high levels of the ALPK1 mutant and its substrates as well as high levels of one or more nucleotide sugar activators of the mutant. This could explain why the diseases are restricted to only a few cell types and why the phenotypes can be variable. Finally, our results raise the possibility of developing selective inhibitors of the disease-causing mutants of ALPK1 by targeting the allosteric ADP-heptose binding site.

## Materials and Methods

### Antibodies.

Antibodies recognising GAPDH (#2118) and anti-rabbit IgG (#7074) were from Cell Signalling Technology and an antibody recognising ALPK1 (#ab236626) was from Abcam.

### DNA Constructs.

The following DNA plasmids encoding FLAG-tagged proteins with expression under the control of the CMV promoter were made by Medical Research Council (MRC) Reagents and Services, MRC Protein Phosphorylation and Ubiquitylation Unit, University of Dundee and are available on request (mrcppureagents.dundee.ac.uk): ALPK1 (DU65668), ALPK1[R153A] (DU71736), ALPK1[R153A/T237M] (DU71739), ALPK1[R153A/Y254C] (DU71737), ALPK1[R153A/V1092A] (DU71738), ALPK1[K233A] (DU71733), ALPK1[K233A/T237M] (DU71768), ALPK1[K233A/Y254C] (DU71764), ALPK1[K233A/V1092A] (DU71735), ALPK1[T237M] (DU65723), ALPK1[T237M/K1067M] (DU71730), ALPK1[Y254C] (DU71685), ALPK1[Y254C/K1067M] (DU71729), ALPK1[K1067M] (DU65680), ALPK1[K1067M/V1092A] (DU71728), ALPK1[V1092A] (DU65703). ALPK1 KO cells were converted to ALPK1/TIFA DKO cells using CRISPR/Cas9 gene-editing technology as described (28) using the guide sequences GAGTAACTTGTCTCCAGATGA (DU64441) and GTGTGTCAGCATCTTCAAAAC (DU64448).

### Nucleotide Sugars and Dinucleotides.

ADP-D,D-heptose (ADP-heptose) and UDP-α-D-mannose were synthesised in-house. ADP-D-ribose (#C7344), cyclic ADP-D-ribose (#A0752), ADP-α-D-glucose (#A0627), GDP-α-D-mannose (#07508), UDP-α-D-glucose (#U4625), UDP-α-D-galactose (#670111), UDP-α-D-glucuronate (#U5625), UDP-N-acetyl-α-D-galactosamine (#U5252), UDP-N-acetyl-α-D-glucosamine (#U4375), NAD (#N7004), and NAD phosphate (NADP) (#N5755) were purchased from Sigma-Aldrich and UDP-α-D-xylose from Carbosource. All nucleotide sugars and dinucleotides were dissolved in PBS to yield a concentration of 1 mM. To stimulate cells, ADP-heptose was added to the culture medium to achieve a final concentration of 5 μM. An equivalent volume of PBS was added to the culture medium in control incubations.

### Protein Kinase Inhibitors.

The IKKβ inhibitor BI605906 (#HY-13019), the TAK1 inhibitor NG25 (#HY-15434), and the JNK inhibitor JNK-IN-8 (#HY-13319) were purchased from MedChemExpress and dissolved in DMSO to yield a concentration of 5 mM. They were added to the culture medium to achieve the final concentrations indicated in figure legends. An equivalent volume of DMSO was added to the culture medium in control incubations.

### Cell Culture, Cell Maintenance and Cell Lysis.

ALPK1 KO HEK293-Blue cells (#hkb-koalpk) and the parental cell line (#hkb-null1v) were purchased from Invivogen and cultured in Dulbecco’s modified Eagle’s medium supplemented with 25 mM 4-(2-hydroxyethyl)-1-piperazineethanesulfonic acid (HEPES) pH 7.3, 10% (v/v) heat-inactivated foetal bovine serum, 100 U/mL penicillin and 0.1 mg/mL streptomycin and maintained at 37 °C in a humidified atmosphere containing 5% CO_2_. For lysis, cells were washed twice with ice-cold PBS and scraped from the plate in lysis buffer containing 50 mM Tris–HCl pH 7.5, 1 mM EDTA, 1 mM ethylene glycol-bis(beta-aminoethyl ether)-N,N,N’,N’-tetracetic acid (EGTA), 1% (v/v) Triton X-100, 1 mM sodium orthovanadate, 50 mM sodium fluoride, 5 mM sodium pyrophosphate, 270 mM sucrose, 10 mM sodium 2-glycerophosphate, 0.2 mM phenylmethylsulfonyl fluoride, and 1 mM benzamidine, supplemented with complete protease inhibitor cocktail. Cell lysates were clarified by centrifugation for 20 min at 20,000 × g at 4 °C and the supernatants (cell extracts) transferred to 1.5 mL microcentrifuge tubes, snap frozen, and stored at –80 °C. The protein concentrations of cell extracts were determined by the Bradford procedure.

### Transfection of HEK293-Blue Cells and Measurement of NF-κB/AP-1 Gene Transcription.

Cells were transfected at 80% confluency using 2.5 µL of lipofectamine 2,000 per 1.0 µg of plasmid, and the culture medium was changed 4 h later. For measurements of SEAP activity in the culture medium, 24-well plates were transfected with 800 ng of plasmid DNA and the cells stimulated with or without ADP-heptose 24 h later. The medium was collected after a further 24 h (unless stated otherwise). Cell culture medium (20 µL) was incubated with 180 µL of QUANTI-blue solution (Invivogen, #rep-qbs), a substrate of SEAP, in a 96-well plate. The plate was incubated at 37 °C for 30 min, and the absorbance at 645 nm was measured using a microplate reader.

To purify ALPK1 for in vitro assays, 60 µg of plasmid DNA encoding WT or mutant ALPK1 was transfected into 15 cm dishes of ALPK1 KO HEK293-Blue cells. After 24 h, the cells were lysed in ice-cold lysis buffer and centrifuged for 20 min (20,000 × g at 4 °C), and the supernatants (cell extracts) were divided into aliquots and stored at –80 °C until immunoprecipitation as described below.

### Immunoprecipitation of FLAG-Tagged ALPK1.

Prior to immunoprecipitation, cell extracts were subjected to immunoblotting to assess the expression level of WT and mutant ALPK1. One hundred micrograms of cell extract protein containing WT FLAG-ALPK1 and normalised amounts of cell extract protein containing the ALPK1 mutants were incubated for 1 h at 4 °C on a rotating wheel with 15 μL of packed anti-FLAG M2 affinity gel (Sigma-Aldrich, #A2220), which had been washed twice with lysis buffer prior to use. After centrifugation for 30 s at 1,000 × g at 4 °C, the supernatant was discarded and the pelleted gel was washed three times with 50 mM Tris-HCl pH 7.5, 1% (v/v) Triton X-100 containing 500 mM NaCl, twice with 50 mM Tris-HCl (pH 7.5), 1% (v/v) Triton X-100, and once with 50 mM Tris–HCl (pH 7.5), 2 mM DTT, 0.1 mM EGTA and 10 mM magnesium acetate.

### Assay of ALPK1 In Vitro.

The immunoprecipitated FLAG-ALPK1 was incubated at 30 °C in 25 µL of 50 mM Tris-HCl (pH 7.5), 2 mM DTT, 0.1 mM EGTA, and 10 mM magnesium acetate containing 8 μM GST-TIFA and 1 mM [γ-^32^P]ATP (specific radioactivity typically 500 cpm/pmol). The reactions were initiated by the addition of [γ-^32^P]ATP and, after the times indicated in figure legends, the reactions were terminated by the addition of LDS sample buffer containing 2.5% (v/v) 2-mercaptoethanol and heated for 5 min at 75 °C. The FLAG resin was pelleted by centrifugation for 30 s at 13,000 × g and the supernatant was subjected to SDS-PAGE. After staining for 30 min with InstantBlue Protein Stain (Abcam, #ab119211) and destaining in water for 16 h with frequent changes, the bands corresponding to GST-TIFA were excised and the incorporation of ^32^P-radioactivity was analysed by Cerenkov counting.

### SDS–PAGE, Transfer to PVDF Membranes and Immunoblotting.

Cell extracts (15 µg of protein) were resolved by sodium dodecyl sulfate polyacrylamide gel electrophoresis (SDS–PAGE), transferred to polyvinylidene fluoride membranes, and analysed by immunoblotting as described previously (3).

## Supplementary Material

Appendix 01 (PDF)

## Data Availability

All study data are included in the article and/or *SI Appendix*.
