# Supplementary material for: ALPK1 mutants causing ROSAH syndrome or Spiradenoma are activated by human nucleotide sugars
Source: Proc Natl Acad Sci U S A. 2023 Dec 7;120(50):e2313148120. doi: 10.1073/pnas.2313148120 (PMC10723048; doi:10.1073/pnas.2313148120)
Supplement: Supplementary file 1 — Appendix 01 (PDF) [file pnas.2313148120.sapp.pdf]

PNAS

Supporting Information for
ALPK1 mutants causing ROSAH syndrome or Spiradenoma are activated by human nucleotide sugars.

Tom Snelling, Anton Saalfrank, Nicola T Wood and Philip Cohen

Philip Cohen
Email: p.cohen@dundee.ac.uk

This PDF file includes:

Figures S1 to S4

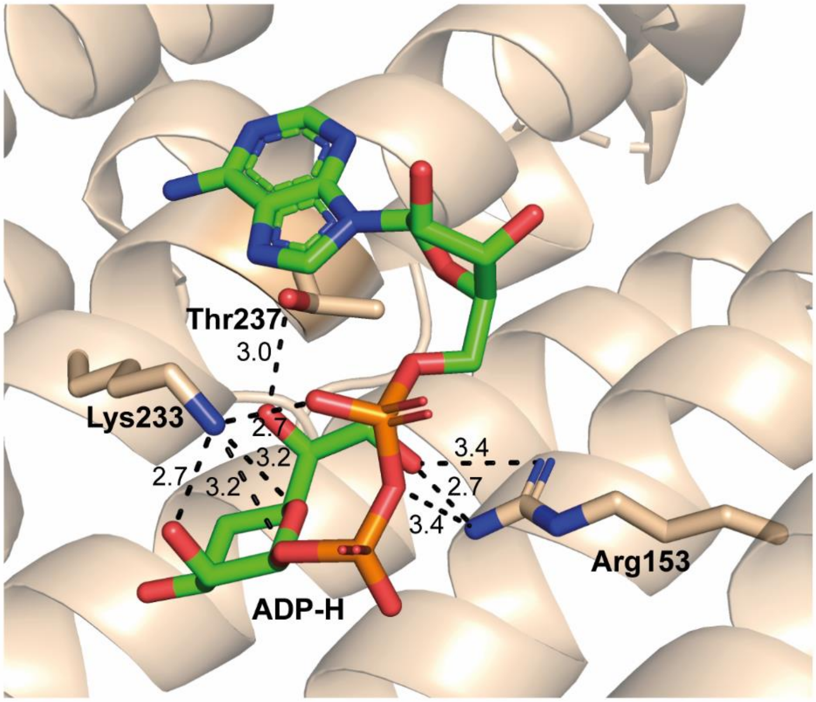

Supplementary Figure 1: Structural analysis of wild-type ALPK1 highlighting the locations of key residues within the ADP-heptose binding site. Cartoon representation of the ADP-heptose binding site of ALPK1 taken from the crystal structure of the ADP-heptose (ADP-H) binding domain (pdb: 5Z2C). Thr237 (which when mutated to Met causes ROSAH syndrome), Arg153 and Lys233 and are shown in stick representation and coloured by element (carbon: bronze; nitrogen: blue; oxygen: red). ADP-H is also shown in stick representation and coloured similarly (except carbon: green; phosphate: orange). The interactions between Thr237, Arg153, Lys233 and ADP-H are shown by black broken lines, with each respective bond length indicated in angstroms.

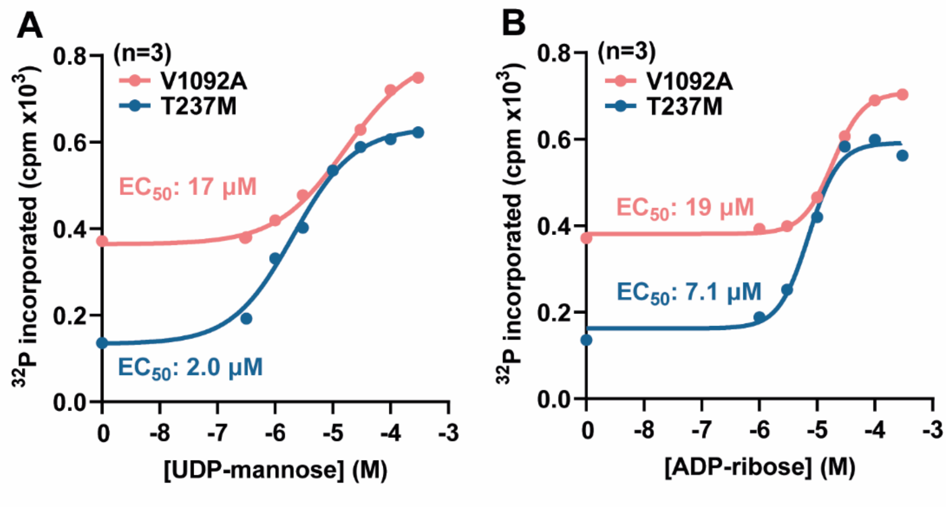

Supplementary Figure 2: Concentrations of UDP-mannose and ADP-ribose required for half-maximal activation of ALPK1[T237M] and ALPK1[V1092A] *in vitro*. (A,B) FLAG-tagged ALPK1[T237M] (T237M, blue bars) or ALPK1[V1092A] (V1092A, pink bars) were immunoprecipitated from cell extracts and assayed for 45 min in the presence of the indicated concentrations of UDP-α-D-mannose (A) or ADP-D-ribose (B) and the incorporation of ^{32}P-radioactivity into GST-TIFA quantified. The experiment was performed in triplicate and the results averaged. The experiment was repeated once more with similar results. The concentrations required for half-maximal activation (EC_{50}) were calculated using non-linear regression in GraphPad Prism (version 9.5.1).

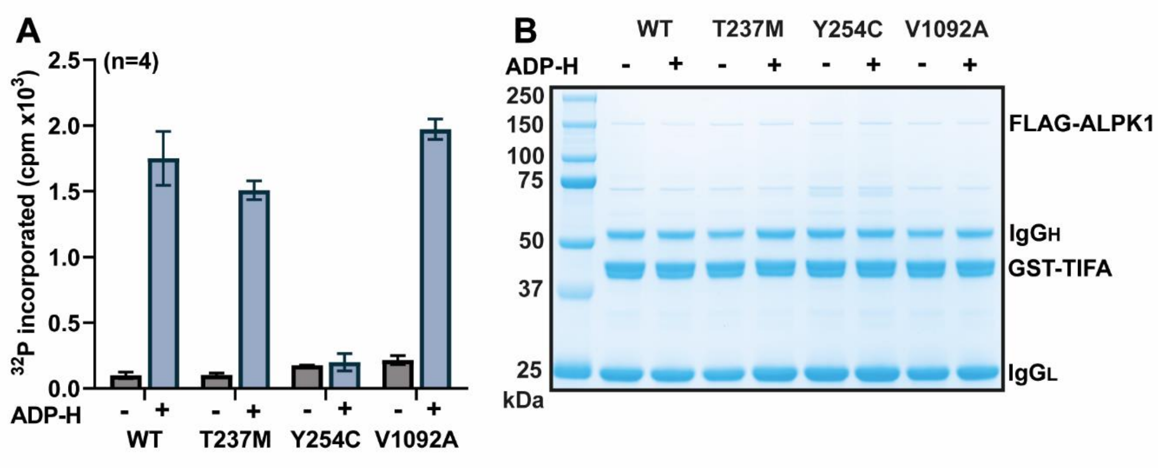

Supplementary Figure 3: **The purified ALPK1[Y254C] mutant is inactive when assayed *in vitro*. (A)** The activities of WT ALPK1 (WT), ALPK1[T237M] (T237M), ALPK1[Y254C] (Y254C) and ALPK1[V1092A] (V1092A) were assayed for 45 min in the absence (gray bars) or presence (blue bars) of 1 µM ADP-heptose (ADP-H). The results are expressed as mean ± SEM from a total of 4 kinase assays (two experiments, each performed in duplicate. (**B**) The reactions from (A) resolved by SDS-PAGE and stained with Coomassie blue, prior to excision of GST-TIFA. The bands corresponding to WT or mutant FLAG-ALPK1, GST-TIFA, immunoglobulin heavy chain (IgGH) and immunoglobulin light chain (IgGL) are indicated.

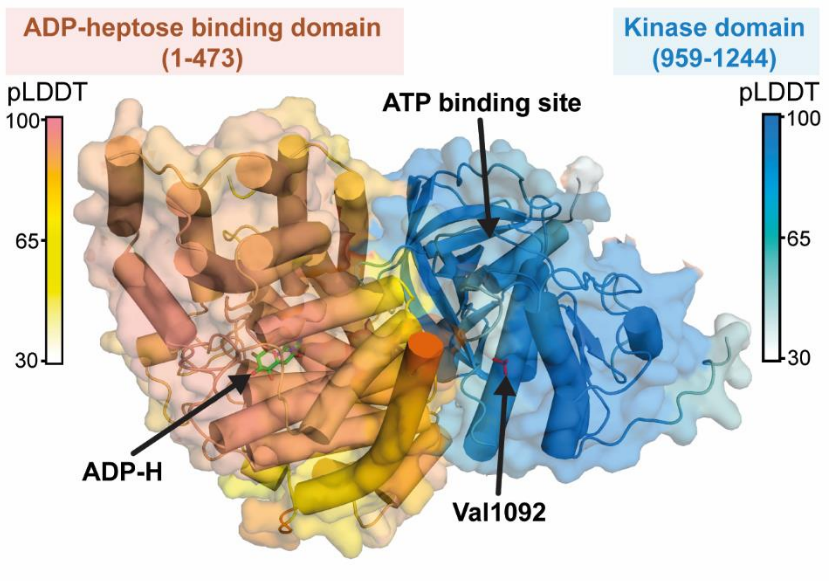

Supplementary Figure 4: AlphaFold2 predicts that the ADP-heptose binding domain of ALPK1 interacts with the catalytic kinase domain of ALPK1. The ADP-heptose (ADP-H) binding domain of ALPK1 (amino acid residues 1-473) and the catalytic kinase domain of ALPK1 (amino acid residues 959-1244) are shown in barrel representation overlaid with a transparent surface representation and are depicted in different colour spectra by the pLDDT scores (from 30 to 100, which indicate the confidence level of the prediction for each residue). The position of the side chain of Val1092 (which when mutated to Ala causes spiradenoma) within the kinase domain is shown in stick representation and coloured red. The ATP binding site (containing Lys1067, which when mutated to Met abolishes enzymatic activity) is also indicated. The position of ADP-H within the ADP-heptose binding domain is shown in stick representation and coloured by element (carbon: green; nitrogen: blue; oxygen: red; phosphate: orange).
